# Traditional herbal treatment induced bilateral amputation of the feet in a five-year-old child: A case report

**DOI:** 10.1016/j.ijscr.2020.04.062

**Published:** 2020-05-11

**Authors:** Komang Agung Irianto, Tri Wahyu Martanto, Rendra Praliestyo Nugroho, Oen Sindrawati, Yudhistira Pradnyan Kloping

**Affiliations:** a Department of Orthopedics and Traumatology, Dr Soetomo General Hospital, Medical Faculty of Universitas Airlangga, Jl. Prof. Dr Moestopo 6-8 Surabaya, 60115, Indonesia; b Pathology Department, Medical Faculty Widya Mandala Catholic University, Indonesia; c Medical Faculty of Universitas Airlangga, Jl. Prof. Dr Moestopo 6-8 Surabaya, 60115, Indonesia

**Keywords:** Bilateral autoamputation, Autoamputation, Necrotizing fasciitis, Herbal treatment, Traditional treatment, Case report

## Abstract

**Introduction:**

Bilateral gangrene of both legs in a child is a rare condition which may lead to sepsis when improperly treated. In certain rare cases, autoamputation may occur instead. We report a case of bilateral symmetrical autoamputation of the feet following necrotizing fasciitis after a fall injury which was treated with herbal medicine in a five-year-old child.

**Presentation of case:**

A five-year-old girl fell from a bicycle and was treated by a traditional healer by wrapping her legs with herbs and leaves. Within 24 h, the skin of both lower limbs darkened. Her vital signs show tachycardia and fever. Physical examination revealed bluish black sharp discoloration of both her lower extremities. On the 3rd day of systemic antibiotic administration, her feet, distal portion of both legs, and part of the thighs darkened progressively. The parents refused any additional intervention due to financial constraints and went back to the traditional healer. A month later, the patient returned to the hospital with both lower extremities autoamputated. The parents denied further treatment. Two months later, the patient was admitted to receive prostheses funded by the city council. Debridement of necrotic bone and soft tissue and skin grafting was performed. In the present day, the patient is in a healthy condition.

**Conclusion:**

The rare occurrence of bilateral autoamputation without any underlying vascular or neurological disorders in this patient is likely caused by vasospasm and thrombosis induced by the herbal treatments which prevent the systemic spread of infection.

## Highlights

• Bilateral gangrene usually progress to a systemic infection and death if not treated properly.
• The development of bilateral gangrene to acute limb ischemia and eventually leading to auto amputation of both feet in this patient was unusual as she did not receive any medical or surgical interventions.
• The rare occurrence of bilateral autoamputation without any underlying vascular or neurological disorders in this patient is likely caused by vasospasm and thrombosis triggered by the herbal treatments, which possibly contain ergot alkaloids, given by the traditional healer.
• This phenomenon may play a part in future considerations for performing amputation to patients with possible necrotizing fasciitis.

## 1 Introduction

Bilateral gangrene of both legs in a child is a severely debilitating condition. If not properly treated, this may progress to a systemic infection and death. In rare reported circumstances, autoamputation may occur. The major cause of bilateral auto-amputation is thrombosis associated with vascular injury [[1], [2], [3]]. In the pathogenesis of thrombosis, infection is the most major factor, followed by trauma and drug reaction [2,3]. Potentially lethal soft tissue infection that develops in a very brief time is a major red flag for physicians [4,5]. Necrotizing fasciitis is one of these red flags, a rare infectious entity in the form of a life-threatening infection, which progresses rapidly through the fascial planes reaching a destruction rate of 2–3 cm/h [6,7]. This is an emergency situation, which can affect an entire extremity within 24 h [5,8]. The incidence has been reported to be 0.08 per 100,000 children per year [5]. This is a problem that physicians in developing countries face, not only in terms of the urgency for diagnosis and treatment but also due to socioeconomic and cultural impact [4]. A Nation-wide population survey conducted in 2014–2015 showed that there is a high prevalence of traditional medicine use among children which is attributed to socioeconomic status, and poor self-rated health status among the country’s rural areas [9]. Irrational traditional healers promising complete recovery through traditional approaches without any scientific basis is one of the main contributors to supposedly preventable complications [10]. We report a case of bilateral symmetrical autoamputation of the feet following necrotizing fasciitis after a fall injury which was treated with herbal medicine in a five-year-old child. This case report has been reported in line with the SCARE criteria [11].

## 2 Case presentation

**Fig. 1:**
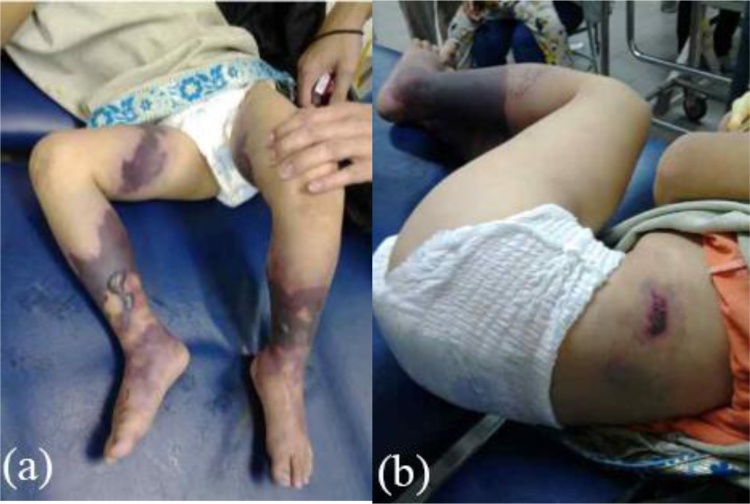
(a), (b) The patient’s lower extremities showing bluish and dark bruises with clear demarcation above the ankles.

**Fig. 2:**
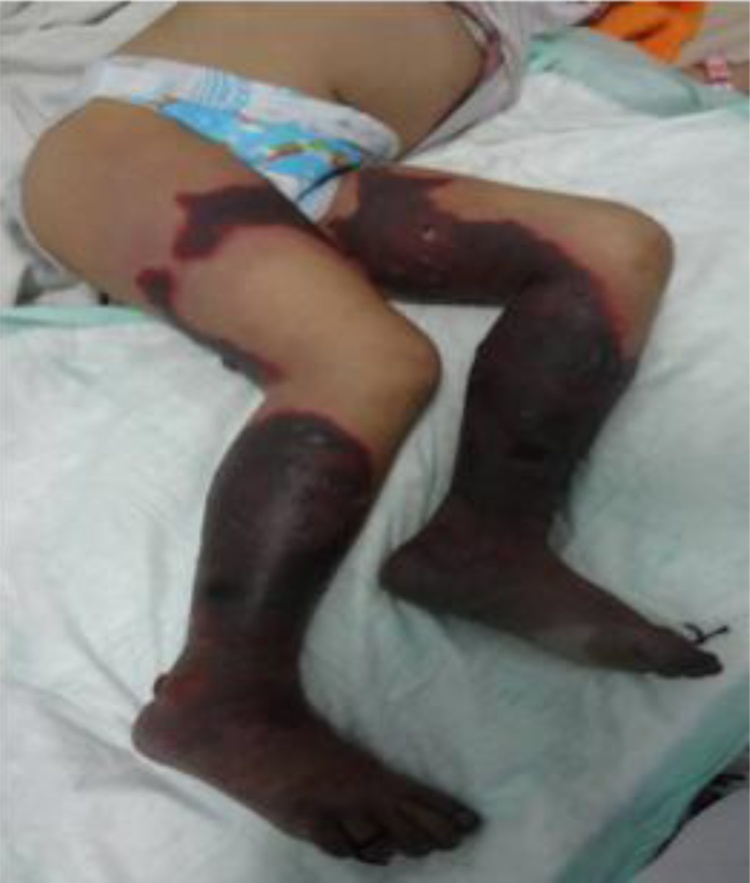
Bilateral necrosis of the patient’s lower extremities involving the pedal and tibial area with some areas of the thighs affected.

**Fig. 3:**
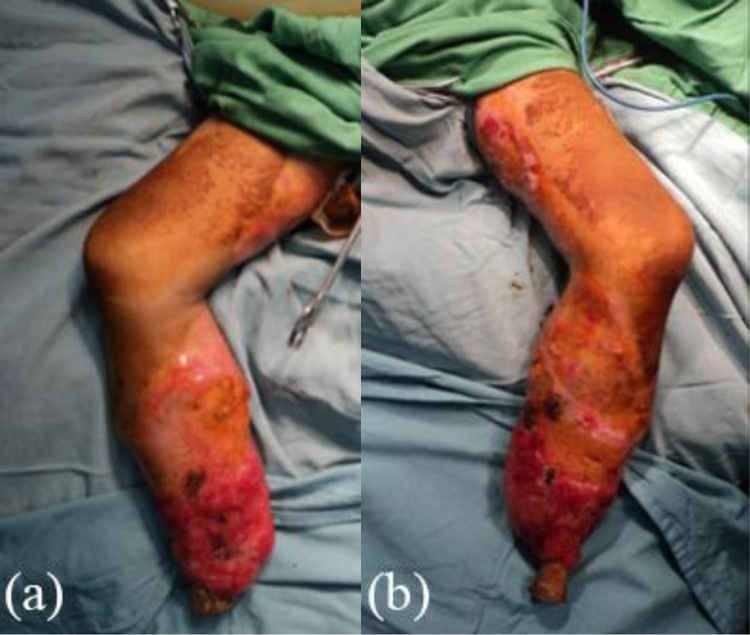
Bilateral Autoamputation of the patient’s lower extremities showing both tibial stumps with exposed bones surrounded by dry necrotic tissue.

**Fig. 4:**
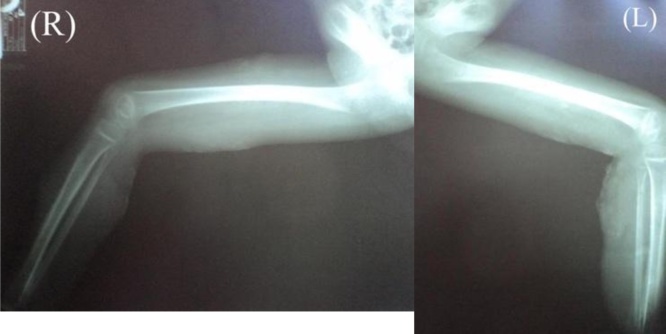
X-Ray of the Patient’s Lower Extremities showing no signs of bone and surrounding inner soft tissue destruction.

**Fig. 5:**
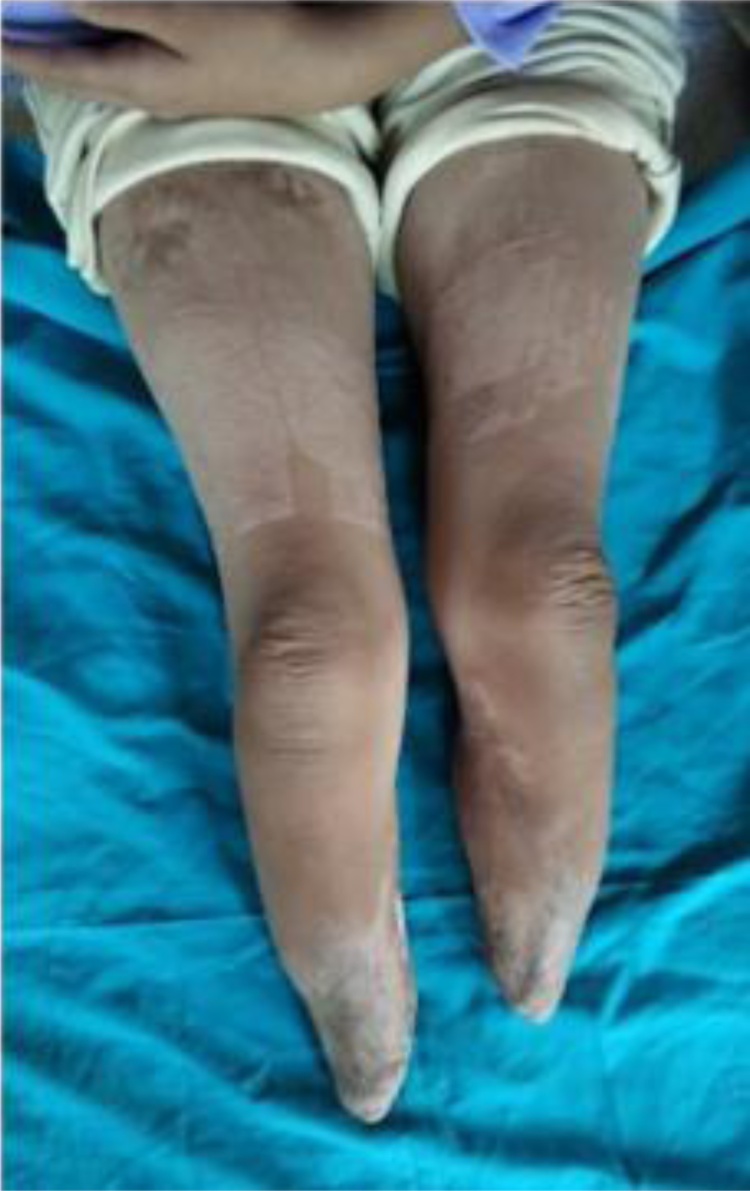
The patient’s Lower Extremities after debridement.

An otherwise healthy five-year-old girl fell from a bicycle and was treated by a traditional healer. According to her parents, at the time she did not have any serious wounds or injuries. Herbs and leaves were applied by the healer to both lower limbs. Within 24 h, the skin of both lower limbs darkened into a bluish and black color with sharp demarcation above the ankles. She was immediately brought to primary care, which referred her to a tertiary private hospital where she was hospitalized with a suspicion of necrotizing fasciitis in both lower limbs. Clinically, the patient was alert and complained that she was feverish and was in intense pain. Vital signs were within normal limits except for her tachycardia (N: 126x/min) and fever (T: 39 °C). Physical examination revealed a bruise in her left lower back as well as bluish black sharp discoloration and demarcation in both distal legs with blisters of the skin. At the time, the sole of her feet was not bluish and was normal in color as shown in Fig. 1. Laboratory results in Table 1 indicated a severe infection. She was treated with broad spectrum antibiotics. Three days after her admission, her feet, distal portion of both legs, and part of the thighs darkened progressively as shown in Fig. 2. Doppler ultrasound examination that the dorsalis pedis arteries pulse were absent. A diagnosis of Purpura gangrenosa vasculitis was made. As the condition of the lower limbs had deteriorated, the parents were explained about the urgent need for bilateral amputation. The parents refused and the parents asked for the patient to be taken home without any further medications. The patient continued her treatment with the traditional healer. The healer believed that wrapping her lags in plastic filled with herbs and leaves would serve as an adequate remedy. A month later, the patient returned to the hospital with both lower extremities amputated at just above the ankle level. Both lower limbs were black and necrotic with dry gangrene from the thighs all the way to the distal. Both distal bone ends were exposed and dry. The patient was alert but looked malnourished. Unfortunately, the parents denied further treatment once again due to financial circumstances. Two months later, the patient was admitted to the surgical ward by the city council to receive prostheses. In spite of the cutaneous gangrene up to the thigh areas, there weren’t any signs of any further propagation of the disease since her last admission. Physical examination showed autoamputation of both legs above the ankles without any signs of sepsis. Both knees were held in a slightly flexed position with adduction of both hips. Fig. 3 shows that both stumps had exposed bone with surrounding necrotic tissue. The lower extremities X-Ray results do not show any signs of destruction of the bones and their surrounding soft tissues as shown in Fig. 4. Debridement of necrotic bone and soft tissue and skin grafting was performed to cover the exposed tissue and bone ends. The child was subsequently fitted with patella tendon bearing prostheses. In the present day, 5 years after the operation, the patient is currently in a healthy condition without any additional complaints and is grateful that she is able to walk again. Her lower extremities do not exhibit any further vascular or neurological abnormalities as shown in Fig. 5.

**Table 1:** Laboratory results during the patient’s admissions.

Laboratory Parameters	Value			
	1st Admission		2nd Admission	3rd Admission
	1st day	3rd day		
Hemoglobin (g/dl)	7.6	7.71	9.2	10.77
Hematocrit (%)	25.2	21.83	–	–
White blood cell count (x/l)	31.000	19.660	5.900	6.290
Platelet (x10^9^/l)	178.000	46.530	458.000	589.000
C-reactive protein (u/l)	41.85	46.1	84.7	2.1
Albumin	3.5	3.6	3.65	2.9
Blood Urea Nitrogen (mmol/l)	11.6	6	–	3
Creatinine Serum (mmol/l)	0.27	0.4	–	0.5
AST (mmol/l)	26	570	–	26
ALT (mmol/l)	12	210	–	10
APTT (second)	>250 (25)	10.9 (K:12)	–	211 (K: 11.8)
PTT (second)	>200 (11)	26.6 (K:25)	–	8.6 (K: 26.7)

## 3 Discussion

During the first admission, the patient’s complaint may be due to improperly treated wounds using topical herbal applications with overlying plastic wrapping by the traditional healer causing infection. The child was first diagnosed with necrotizing fasciitis as he was presented with severe skin and soft tissue infection through the fascial planes with extensive involvement and marked skin necrosis [12]. Referring to the LRINEC (Laboratory Risk Indicator for Necrotizing fasciitis) score, the findings of the white blood cell count (>25,000) and Hemoglobin level (<11 g/dl) result in a total score of 4 (<5) indicating a low risk [13]. However, it is unusual for an infection to spread so rapidly leading to necrosis of the skin and soft tissue. Based on the speed of soft tissue infection and destruction, this appeared to be the fulminant type of necrotizing fasciitis [14]. In this case, the patient suffered greatly from pain and fever, but her consciousness was intact. In order to reduce potential morbidity and mortality, suspecting a severe soft tissue infection as necrotizing fasciitis is recommended as soon as there are skin color changes with demarcation [7]. The surgeon should not wait for all the laboratory results or deterioration of the patient’s condition since this is a potentially lethal soft tissue infection which requires prompt intervention. Just before the debridement the patient’s both lower legs were already blue black, including the feet which were normal before as shown in Fig. 2. Both dorsalis pedis pulses were absent indicating signs of acute limb ischemia [15]. As a result, the decision swung towards amputation, which the patient’s family refused in favor of a traditional alternative due to financial constraints. The eventual development of bilateral acute limb ischemia leading to auto-amputation of both feet in this patient was also unusual. The ability of the child to endure severe soft tissue infection with loss of both lower limbs without succumbing to sepsis without any antibiotic administration was unexpected. There are several publications from Africa regarding symmetrical bilateral peripheral gangrene due to unknown etiology called Tropical Idiopathic Lower Limb Gangrene [2,3,16,17]. Many of these reported cases exhibited similar findings to this patient. One of the publications reported a patient with sudden onset of leg pain followed by bilateral lower limb gangrene two days after traditional herbal treatment. The patient survived after bilateral below knee amputations [16]. Similar findings are also reported in a case series of peripheral gangrene in African children, involving the application of traditional herbs and leaves on the limbs, without history of major trauma. In the 12 cases reported, seven cases reported normal coagulation tests, whereas the other two cases showed absence of thrombi in the amputated limbs, yet extreme vasospasm was found [17]. These findings were suspected to be linked to alpha receptor stimulating drugs with strong vasospasm effect like ergotamine. Ergot-like alkaloids can be found in many herbs and traditional roots. Even though we were not able to identify the herbs in the patient, based on the clinical findings the possibility is very likely [1]. Most Indonesian traditional herbs contain ergot-like alkaloids [18]. The possibility of extreme vasospasm in this case could explain why dry gangrene occurred after initially presenting with wet gangrene. When the child was first admitted, her WBC was very high. After antibiotic administration, the level decreased on the third day indicating a response to the treatment. However, the low platelet count on day three was worrisome. The tissue necrosis process persisted, indicated by high CRP and high increase of serum transaminase (ALT) levels. The laboratory results in Table 1 showed impairment of the coagulation system indicated by high PTT/APTT levels which dropped to normal levels over a three-day period associated with thrombocytopenia suggesting the possibility that the platelet was depleted due the formation of thrombi [15,19,20]. This pathophysiology process was reported by Wakhlu et al. who performed delayed debridement two to four days later. This would result in minimal blood loss; thus, the debridement could be carried out by peeling off the darkened and dry tissue leaving the fresh granulation tissue beneath without the need for anesthesia [21]. This management of necrotizing fasciitis is based on the theory that microthrombi in the subcutaneous vessels automatically barred further in and out flow, thus preventing any further propagation of local or systemic infection. It is possible that in this patient, formed microthrombi prevented the further spread of infection, hence resulting in a dry gangrene leading eventually to a bilateral autoamputation as shown in Fig. 3, Fig. 4.

## 4 Conclusion

The rare occurrence of bilateral autoamputation without any underlying vascular or neurological disorders in this patient is likely caused by vasospasm and thrombosis triggered by the herbal treatments given by the traditional healer. Nevertheless, the vasospasm and thrombi prevented the spread of infection and may have saved the patient’s life by limiting the extend to the lower extremities. This phenomenon may play a part in future considerations for performing amputation to patients with possible necrotizing fasciitis.

## Declaration of Competing Interest

The authors declared no potential conflicts of interest with respect to the research, authorship and/or publication of this article.

## Sources of funding

The authors received no financial support for the research, authorship and/or publication of this article.

## Ethical approval

Ethical approval to report this case was obtained from The Hospital Research Ethics Committee of “*RSUD Dr. Soetomo*” where the patient was admitted.

## Consent

The patient’s parents provided written informed consent granting permission for patient information and images to be published anonymously.

## Authors contribution

**Komang Agung Irianto**: Conceptualization, Investigation, Resources, Writing – Original Draft, Supervision, Project Administration.

**Tri Wahyu Martanto**: Investigation, Resources, Writing – Original Draft.

**Rendra Praliestyo Nugroho**: Investigation, Resources, Writing – Original Draft.

**Oen Sindrawati**: Investigation, Resources, Writing – Original Draft.

**Yudhistira Pradnyan Kloping**: Conceptualization, Writing – Original Draft, Writing – Review and Editing.

## Registration of research studies

NA.

## Guarantor

Komang Agung Irianto.

## Provenance and peer review

Not commissioned, externally peer-reviewed.
